# Patient-Reported Safety Events in Chronic Kidney Disease Recorded With an Interactive Voice-Inquiry Dial-Response System: Monthly Report Analysis

**DOI:** 10.2196/jmir.5203

**Published:** 2016-05-26

**Authors:** Jeffrey C Fink, Rebecca M Doerfler, Marni R Yoffe, Clarissa J Diamantidis, Jacob B Blumenthal, Tariq Siddiqui, James F Gardner, Soren Snitker, Min Zhan

**Affiliations:** ^1^ University of Maryland School of Medicine Department of Medicine Baltimore, MD United States; ^2^ Duke University School of Medicine Department of Medicine Durham, NC United States; ^3^ University of Maryland School of Pharmacy Pharmaceutical Research Computing Baltimore, MD United States; ^4^ University of Maryland Department of Epidemiology and Public Health Baltimore, MD United States

**Keywords:** patient-reported outcomes, CKD, interactive voice-response system, patient safety

## Abstract

**Background:**

Monitoring patient-reported outcomes (PROs) may improve safety of chronic kidney disease (CKD) patients.

**Objective:**

Evaluate the performance of an interactive voice-inquiry dial-response system (IVRDS) in detecting CKD-pertinent adverse safety events outside of the clinical environment and compare the incidence of events using the IVDRS to that detected by paper diary.

**Methods:**

This was a 6-month study of Stage III-V CKD patients in the Safe Kidney Care (SKC) study. Participants crossed over from a paper diary to the IVDRS for recording patient-reported safety events defined as symptoms or events attributable to medications or care. The IVDRS was adapted from the SKC paper diary to record event frequency and remediation. Participants were auto-called weekly and permitted to self-initiate calls. Monthly reports were reviewed by two physician adjudicators for their clinical significance.

**Results:**

52 participants were followed over a total of 1384 weeks. 28 out of 52 participants (54%) reported events using the IVDRS versus 8 out of 52 (15%) with the paper diary; hypoglycemia was the most common event for both methods. All IVDRS menu options were selected at least once except for confusion and rash. Events were reported on 121 calls, with 8 calls reporting event remediation by ambulance or emergency room (ER) visit. The event rate with the IVDRS and paper diary, with and without hypoglycemia, was 26.7 versus 4.7 and 18.3 versus 0.8 per 100 person weeks, respectively (*P*=.002 and *P*<.001). The frequent users (ie, >10 events) largely differed by method, and event rates excluding the most frequent user of each were 16.9 versus 2.5 per 100 person weeks, respectively (*P*<.001). Adjudicators found approximately half the 80 reports clinically significant, with about a quarter judged as actionable. Hypoglycemia was often associated with additional reports of fatigue and falling. Participants expressed favorable satisfaction with the IVDRS.

**Conclusions:**

Use of the IVDRS among CKD patients reveals a high frequency of clinically significant safety events and has the potential to be used as an important supplement to clinical care for improving patient safety.

## Introduction

Recording patient-reported outcomes (PROs) is important for effective management of chronic disease management. The US Food and Drug Administration (FDA) defined a PRO as a report of health status coming directly from a patient “without amendment or interpretation by a clinician” [1]. The nephrology community recognizes the need to monitor PROs in chronic kidney disease (CKD) care [2,3]. Areas of emphasis to date include assessing dialysis patients’ physical and mental impairment [4], preferences and experiences with renal replacement therapy [5,6], and the symptom burden of CKD-related anemia [7]. General tools for documenting PROs, such as the 36- and 12-item Short Form Health Survey (SF-36 and SF-12,respectively), are used in kidney disease; however, kidney disease PRO assessments are more commonly applied in the research domain than in the clinic [8]. Tools to record clinically significant and actionable CKD PROs are needed [2].

Monitoring and interpreting PROs may give insight into means of reducing patient safety events, here defined as harm from medical therapy [9-11]. Presenting underdetected PROs to providers, especially medication-related adverse events, can offer new opportunities to improve the safety of delivered care. Patient diaries have been employed to record PROs related to medication tolerance in clinical trials as well as with disease management, and both paper and electronic mediums have been used [12,13]. CKD patients have multiple comorbidities, take numerous medications, often report safety-related outcomes and experiences [14], and may benefit from monitoring such PROs.

In this study, we examined a subcohort of predialysis CKD patients in the Safe Kidney Care (SKC) study (NCT01407367). We used an interactive voice-inquiry dial-response system (IVDRS) to gauge patient-reported experiences attributed to medications and considered adverse safety events. We demonstrate the participants’ usage of the IVDRS relative to the SKC paper diary, report the serial trends in engagement, tabulate the types of events reported, evaluate the clinical importance of the reported events, and survey participant satisfaction with the reporting system.

## Methods

### Study Overview

The IVDRS study is an ancillary to the ongoing SKC cohort study with the latter commencing in 2011. The protocol and informed consent were approved by the University of Maryland Institutional Review Board. The SKC study tracked CKD patients through annual in-center visits to determine the frequency of an array of adverse safety events. The study included two subgroups: Phase 1 participants were provided with a medical alert accessory to augment kidney disease awareness and were given access to the SKC website providing best practices in safe CKD care; Phase 2 SKC participants received no accessory or website access, but were followed on the same schedule as Phase 1 participants for detection of adverse safety events. SKC monitoring included provision of a paper diary to document adverse events that the participant attributed to a medication, medical instructions, or medical care. The structured check-off entries in the SKC diary were predetermined after an online nephrologist survey and an expert panel reviewed, adjudicated, and categorized prominent adverse safety events. The diary also permitted text entries not included in the menu of structured events. Participants were instructed to mail in diary pages—using study-issued postage-paid envelopes—with documented entries and, as per the protocol, were reminded every 3 months to use their diary. Text entries were reviewed semiannually to determine if they could be reclassified into a structured entry or if they warranted a unique adverse safety event category.

The IVDRS platform was a Linux-based operating system provided and administered by CircleLink Health (Stamford, CT). The phone-based interactive modality uses the telephone dial-pad for participant responses to automated voice queries. The IVDRS query menu is derived from the SKC paper diary using a similarly structured list of adverse safety events, but with each adverse safety event linked to a series of more-detailed questions, including event frequency and determination of the action taken for each event (eg, “Did you feel dizzy in the last 7 days? Press ‘1’ for yes or ‘2’ for no.” “Enter the number of times you felt dizzy. Was the dizziness caused by a medicine? Press ‘1’ for yes or ‘2’ for no.” “How did you treat your dizziness? Press ‘1’ if you called an ambulance or went to the emergency room. ‘2’ if you called your doctor. ‘3’ if you self-treated. ‘4’ if you did nothing. ‘5’ if you did more than one of these.”). The IVDRS query for hypoglycemia included a threshold of less than 70 mg/dL versus 60 mg/dL by paper diary. The more stringent threshold with the latter was based on the expectation that participants would be less inclined to mail in entries for less severe hypoglycemia. Given the number of potential adverse safety events, the first menu of dial options included what was expected to be the most frequent events along with an option to proceed to a second menu for the remaining adverse safety events to select if they occurred. An option was also included to dial in if no events occurred. The IVDRS protocol was programmed to call study participants weekly and, once calls were answered, the IVDRS queried participants about events during that day and the preceding 6 days. If a given weekly call was unanswered, the system would call again 15 minutes later, but if there was no answer on the second call, then the IVDRS ceased calling until the next week. The IVDRS also had the capability for participants to initiate calls for events they deemed necessary to document prior to the scheduled weekly call with an associated time stamp. If the participant elected to use the call-in option within 24 hours of the scheduled weekly call, the latter was not initiated.

### Study Participants and Baseline Assessment

The IVDRS study enrolled 52 consecutive consenting SKC participants between June 2, 2014, and December 18, 2014, at any postbaseline annual study visit, without regard to the frequency of paper diary use. Participants without a stable telephone number—cellular or ground line—or who were expected to reach dialysis or die over the next 6 months were not enrolled in the IVDRS study. Information collected from the core SKC study visit when the participant was recruited included the following: in-center vital signs, serum 6-8-hour fasting glucose, potassium, creatinine for estimation of glomerular filtration rate (GFR) and venous hemoglobin, and the annual medications reported. Also, paper diary pages from the prior 6 months were reviewed and events were documented.

Each consenting participant was asked for a preferred weekly IVDRS call time over the 6 months of the protocol. Each participant was instructed on the use of the IVDRS system with a mock IVDRS interaction and was told that events to be submitted were those they believed to be attributable to a medicine or their medical care, similar to the SKC paper diary protocol. At the completion of the protocol, each participant returned to the study center for an update of reported medications, medical event update, and satisfaction survey.

IVDRS data was received by the vendor and transmitted to the study team via a Web-based portal with daily coordinator review for obvious data entry errors; urgent adverse safety events (eg,  ≥ 1 reported fall) were flagged and promptly reviewed. A monthly report was prepared for each participant with all events detected via weekly calls or self-initiated calls in a 4-week window. Monthly reports along with the SKC visit vital sign readings, laboratory values, medical comorbidities, and medications were presented to two independent physician reviewers (JBB, CJD) for determination of whether each report was (1) of no interest, (2) of interest, but with no action recommended, or (3) of interest with action recommended. A separate physician reviewer (SS) examined each adverse safety event to determine if participants with adverse events had reported taking a medication that could plausibly cause such an experience based on attributable medication categories previously defined [14].

### Analysis

The analysis was designed to be descriptive with demographic characteristics of the IVDRS participants reported as means (SD) and with n (%) for categorical variables. The IVDRS patient-reported adverse safety events were compared to the SKC paper diary patient reports as a baseline reference and using the generalized estimating equation (GEE) with Poisson distribution and a log link function. IVDRS and SKC paper diary event rates were reported per 100 participant weeks; IVDRS adverse events were also presented by months of participation and by adverse safety event category. The final visit was 26 ± 2 weeks in length to accommodate participants’ schedules. Association (ie, market basket) analysis was also employed to identify the likelihood of coexistent adverse safety event types by participant using the IVDRS and treating the entire study period as a single observation period. Association analysis measures included confidence, which indicates the likelihood of a consequent event given the occurrence of an antecedent event, and the lift ratio, which is the confidence over the prevalence of the consequent event independent of the antecedent. A lift ratio of 1 indicated that the co-occurrence of events was random [14-16]. Analyses were conducted in SAS version 9.3 (SAS Institute, Inc; Cary, NC).

## Results

### Characteristics of Study Participants

The characteristics of the 52 participants are shown in Table 1. Almost 60% of participants were greater than 65 years of age and a majority were male and African American. Approximately two-thirds of participants had Stage III-B through Stage V CKD at the time of enrollment, most had diabetes, and about half had previously used the Internet. The averages of study participants’ clinical measures were in acceptable ranges; however, there was substantial polypharmacy. All study participants completed the study protocol, except for one who died after completing 22 weeks of the 6-month protocol.

**Table 1:** Characteristics of interactive voice-inquiry dial-response system (IVDRS) study participants.

Participant characteristics		Participants (n=52) or values, n (%), mean (SD), or median (Interquartile range)
**Time in Safe Kidney Care study, n (%)**		
	1 year	21 (40)
	2 years	14 (27)
	3 years	17 (33)
**Age in years, n (%)**		
	≤ 60	10 (19)
	61-65	11 (21)
	66-70	18 (35)
	≥71	13 (25)
**Gender, n (%)**		
	Male	40 (77)
	Female	12 (23)
**African American, n (%)**		
	Yes	39 (75)
	No	13 (25)
**Systolic blood pressure (mm Hg), mean (SD)**		
	Sitting	134 (20.6)
	Standing, (n=50), 2 participants could not stand	134 (22.4)
**Diastolic blood pressure (mm Hg), mean (SD)**		
	Sitting	70 (12.6)
	Standing (n=50), 2 participants could not stand	72 (15.5)
**Glomerular filtration rate (ml/min/1.73m**^2^ **), n (%)**		
	≤ 45	34 (65)
	> 45	18 (35)
Serum potassium (meq/L), mean (SD)		4.4 (0.6)
Serum glucose (mg/dL), mean (SD)		136.3 (57.4)
Venous hemoglobin (g/dL), mean (SD)		11.9 (1.6)
**Cardiovascular disease, n (%)**		
	Yes	23 (44)
	No	29 (56)
**Cancer, n (%)**		
	Yes	17 (33)
	No	35 (67)
**Diabetes, n (%)**		
	Yes	47 (90)
	No	5 (10)
**Used the Internet in the past year to look for health information, n (%)**		
	Yes	23 (44)
	No	29 (56)
Number of medications, median (IQR)		16 (10.0)

### Use of the Interactive Voice-Inquiry Dial-Response System

Table 2 shows study participation over 1384 weeks distributed by reporting any or no safety events and further classified by expected and detected weeks of participation. Table 2 also tabulates call response by frequency of events per call and participant remediation of events reported on each call. A total of 24 out of 52 (46%) study participants reported no events over 650 weeks of participation, including 619 weeks when a call was delivered or self-initiated. A total of 28 out of 52 (54%) study participants reported at least one safety event over 734 total weeks of participation, of which 731 had calls delivered or self-initiated. Events were reported in 113 weeks with a total of 121 calls either delivered or self-initiated with one or more reported events. Most calls included one event and self-treatment, but a notable minority (8/121, 6.6%) involved an ambulance or emergency room (ER) visit.

**Figure 1:**
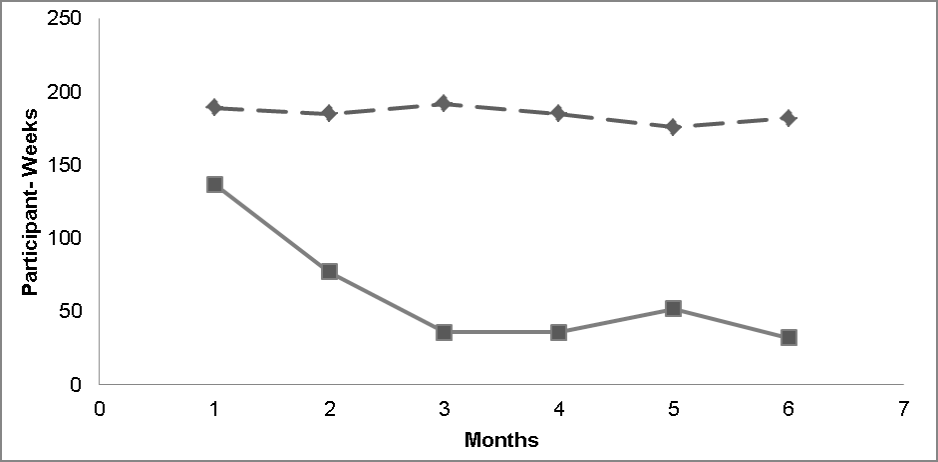
Participant weeks with study participation and adverse event reporting by month. The dashed line shows the trend in the number of active participant weeks per month—an active week is defined as one or more dialed entry, including “no events to report”—and the solid line represents total number of participant weeks with safety events per month.

Figure 1 shows the trend in monthly study engagement along with event reporting. The dashed line shows the trend in the number of active participant weeks per month—an active week is defined as one or more dialed entry, including “no events to report”—and the solid line represents total number of participant weeks with safety events per month. While the frequency of event reporting declined over the study, the IVDRS engagement measured by active participant weeks remained relatively constant.

Only 10 out of 1227 (0.81%) calls with participant entries were found to be erroneous after coordinator-initiated call inspection or by system alert for extreme values. In such instances, staff contacted participants and database corrections were made after clarification. A total of 31 out of 1227 (2.53%) initial entries had errors that were self-corrected during the call when the participant was prompted to confirm his/her selection.

**Table 2:** Distribution of interactive voice-inquiry dial-response system activity by participants, weeks of participation, and events.

Participant type			Number of participants (n=52), n (%)	Weeks of participation (n=1384), n (%)	Weeks with delivered or self-initiated calls (n=1350), n (%)	Calls with events reported (n=121)^a^, n: number of events or remediation	
						Calls by number of events	Calls by associated remediation
**With no events**			24 (46)				
	No call			31 (2.24)			
	**Call delivered or self-initiated**			619 (44.73)			
		Not picked up			80 (5.93)		
		No events reported			539 (39.93)		
**With events**			28 (54)				
	No call			3 (0.22)			
	**Call delivered or self-initiated**			731 (52.82)			
		Not picked up			138 (10.22)		
		No events reported			480 (35.56)		
		Events reported			113 (8.37)		
		Event details				115: 1 event 5: 2 events 1: 3 events	8: Ambulance/ER^b^ visit 3: called MD^c^ 90: self-treated 14: nothing done 4: multiple actions 1: hung up

^a^Participants may self-initiate a call more than once a week.

^b^ER: emergency room.

^c^MD: medical doctor.

### Patient-Reported Safety Events Using the Interactive Voice-Inquiry Dial-Response System Relative to the Paper Diary

Table 3 shows that the number of reported events was significantly greater with the IVDRS versus the paper diary. In the 6 months preceding IVDRS enrollment, participants submitted 85 paper diary entries with 95 patient-reported events. Of those 95 events, 39 (41%) patient-reported symptoms were text entries, with 3 out of 39 (8%) classified into existing categories and 5 out of 39 (13%) reports of feeling sick or having bruises, which were classified as other adverse safety events. A total of 31 out of 95 (33%) text entries were for pain and were considered as not meeting the criteria of patient-reported safety events (ie, not related to medical care). The exclusion of these entries reduced the total number of patient-reported safety events to 64.

**Table 3:** Comparison of adverse safety events detected by the Safe Kidney Care paper diary versus the interactive voice-inquiry dial-response system (IVDRS).

Participant categories		Paper diary, n or n (participant [P] identifier)	IVDRS^a^, n or n (participant [P] identifier)
Total weeks of participation^b^		1352	1384
Participants with events		8	28
Participants with no events		44	24
Total safety events		64	370
Maximum safety events per participant		32	101
Total safety events excluding highest reporter		32	225
Total events excluding hypoglycemia		11	253
Events per 100 participant weeks		4.7	26.7^c^
Events per 100 participant weeks excluding highest user		2.5	16.9^d^
Events per 100 participant weeks excluding hypoglycemia		0.8	18.3^d^
**Top reporters and number of events,** **n (participant [P] identifier)**			
	1	32 [P5]	101 [P33]
	2	12 [P3]	45 [P27]
	3	10 [P29]	44 [P5]
	4	5 [P50]	32 [P20]
	5	2 [P4]	28 [P41]
	6	1 [P1, P9, P10]	26 [P50]
**Top safety events reported**			
	Hypoglycemia	53	117
	Leg swelling	1	80
	Dizziness	1	77
	Falling	0	12
	Bleeding	0	1
	Other	9	83

^a^IVDRS: interactive voice-inquiry and dial-response system.

^b^One participant did not complete the study but contributed 22 weeks.

^c^ *P*=.002.

^d^ *P*<.001.

With both modalities, hypoglycemia was the most common safety event, but the number of reported hypoglycemic events was greater with the IVDRS than with the paper diary. When excluding the most frequent reporter of adverse safety events with each modality, the frequency of reporting with IVDRS remained significantly higher. Similarly, when excluding hypoglycemia, the IVDRS modality had a significantly higher rate of reported events. Table 3 also displays the top 6 reporters by modality and demonstrates that 4 frequent users in each modality were not among the top-ranked users with the other modality.

Table 4 reveals the distribution of all 370 events reported via the IVDRS by study month. Hypoglycemia represented 31.6% of events across the study period, followed by leg or ankle swelling, dizziness, and fatigue. Of note, only one participant reported events that were not conditional on a potentially attributable medication. Omitting those events not meeting this conditional criterion for a safety event reduced the total number of events from 370 to 356.

**Table 4:** Distribution of patient-reported adverse safety events using the interactive voice-inquiry dial-response system (IVDRS) over the study period.

Symptoms	Patient-reported adverse safety events, n (%)						
	Months						
	1 (n=137)	2 (n=77)	3 (n=36)	4 (n=36)	5 (n=52)	≥6 (n=32)	Total (n=370)
Total symptoms (n=370)	137	77	36	36	52	32	370
Low blood sugar	27 (19.7)	26 (34)	15 (42)	24 (67)	10 (19)	15 (47)	117 (31.6)
Leg or ankle swelling	27 (19.7)	29 (38)	11 (31)	0 (0)	12 (23)	1 (3)	80 (21.6)
Face swelling	0 (0)	7 (9)	0 (0)	0 (0)	0 (0)	0 (0)	7 (1.9)
Dizziness	28 (20.4)	5 (6)	0 (0)	0 (0)	28 (54)	16 (48)	77 (20.8)
Fall	1 (0.7)	0 (0)	3 (8)	7 (19)	1 (2)	0 (0)	12 (3.2)
Stomach problems	7 (5.1)	2 (3)	0 (0)	0 (0)	0 (0)	0 (0)	9 (2.4)
Bleeding	0 (0)	0 (0)	0 (0)	0 (0)	1 (2)	0 (0)	1 (0.3)
High potassium	1 (0.7)	1 (1)	0 (0)	0 (0)	0 (0)	0 (0)	2 (0.5)
Skin rash	0 (0)	0 (0)	0 (0)	0 (0)	0 (0)	0 (0)	0 (0)
Confusion	0 (0)	0 (0)	0 (0)	0 (0)	0 (0)	0 (0)	0 (0)
Fatigue	46 (33.6)	7 (9)	7 (19)	0 (0)	0 (0)	0 (0)	60 (16.2)
Low blood pressure	0 (0)	0 (0)	0 (0)	5 (14)	0 (0)	0 (0)	5 (1.4)

With 23 of 52 participants (44%) reporting more than one event during the study period, we found the strongest association of hypoglycemia with fatigue and falling. Participants who reported fatigue had 66.6% confidence of reporting an episode of hypoglycemia at some time during the study period, with a lift ratio of 2.31. Likewise, participants who reported a fall had 50% confidence of reporting a hypoglycemic episode resulting in a lift ratio of 1.73.

### Clinical Significance of Patient-Reported Safety Events

A significant proportion of patient-reported safety events were judged to be clinically important by the pair of physician adjudicators reviewing participants’ cumulative monthly reports of all IVDRS-reported events, in conjunction with SKC-measured laboratory values—estimated GFR (eGFR), potassium, hemoglobin, and fasting glucose—reported comorbidities, and medications collected on study entry. Of the 80 reports reviewed, the two adjudicators individually found 20.0% and 11.3% of them of no clinical interest, 53.8% and 46.3% of them of clinical interest but no action would be taken, and 26.3% and 42.5% of them warranting of clinical action.

### Participant Feedback and Satisfaction With the Interactive Voice-Inquiry Dial-Response System

At completion of the study, participants reported a universally high degree of satisfaction with the IVDRS. Table 5 shows that more than 90% of the participants agreed or strongly agreed that the IVDRS was easy to use and was used confidently and weekly. Most participants liked using the IVDRS and would recommend its use to other CKD patients in the future.

**Table 5:** Participant satisfaction with interactive voice-inquiry and dial-response system (IVDRS) use.

Satisfaction survey items	Participant response (n=51^a^), n (%)						
	1 (strongly agree)	2	3	4 (neutral)	5	6	7 (strongly disagree)
It is easy to use the eDiary to record safety events that happen to me	22 (43)	28 (55)	0 (0)	1 (2)	0 (0)	0 (0)	0 (0)
I would recommend the eDiary service to other people with kidney problems	22 (43)	27 (53)	0 (0)	1 (2)	0 (0)	1 (2)	0 (0)
I like using the eDiary to record safety events that happen to me	20 (39)	27 (53)	1 (2)	1 (2)	0 (0)	2 (4)	0 (0)
I think that I would like to use this service often to report medical safety events to my doctors	20 (39)	27 (53)	0 (0)	1 (2)	0 (0)	3 (6)	0 (0)
I like when the system called me on a weekly basis to ask me to record medical safety events	18 (35)	30 (59)	0 (0)	1 (2)	1 (2)	1 (2)	0 (0)
I felt very confident using the eDiary	17 (33)	31 (61)	1 (2)	0 (0)	1 (2)	1 (2)	0 (0)
I felt that the eDiary system was hard to use	0 (0)	0 (0)	1 (2)	0 (0)	0 (0)	37 (73)	13 (26)

^a^One participant died before completion of the protocol.

## Discussion

### Principal Findings

PROs can provide important information on CKD-related adverse safety events outside the medical care setting. Such experiences may be overlooked or underreported because they are not recalled by patients or solicited by providers; moreover, patients may have difficulty in drawing associations between such events and their treatments. This study demonstrates the utility of an IVDRS in monitoring a dimension of the patient experience that can be related to safe care. The application of this elementary form of remote data capture of CKD patient-reported safety incidents reveals a high rate of events in the context of potentially attributable medication usage. The number of events captured by the IVDRS is high relative to a paper diary used by the same participants. A notable number of the reported events were found to be significant as they led to urgent responses, while conversely, a high frequency of these PROs were self-treated and perhaps underappreciated by the reporters for their clinical significance. Independent review of these events revealed a substantial proportion of them to be important and actionable given the clinical context in which they occurred. Participants were favorable in their review of the system and provided encouraging evidence that this common household technology can be used to enhance chronic disease management in CKD [17].

### Limitations

The study has inherent limitations to be considered when interpreting the results. The completeness of data collected using the IVDRS to record safety events is limited by participants’ motivation in using the system. We attempted to minimize potential reporting bias based on severity or frequency of events by scheduling weekly calls and limiting participant dependence on memory. The relatively short time frame between calls was intended to improve recollection of events across a wider severity range and avoid restricting the selection of only the most significant of events.

While the comparisons between the IVDRS and paper diary means of event recording are paired within participants, the modalities have inherent differences that must be acknowledged when contrasting event rates. The scheduled contact of participants by the IVDRS effectively solicits responses in comparison to the expected self-initiated use of the paper diary—even with 3-month reminders—and most likely enhances the detection of adverse events. The IVDRS menu of delivered questions may compel the participant to consider more thoroughly their safety experiences over the duration of the study. Additionally, the higher threshold set with the IVDRS for incidence of hypoglycemia may also trigger a higher reporting rate relative to the paper diary. However, examining the frequency of events with each modality is still informative in demonstrating the extent to which the IVDRS detects a wide range and high frequency of adverse safety events, even when excluding hypoglycemia.

The IVDRS protocol resulted in an apparent time-dependent decline in patient-reported safety events. While the secular fall-off in event reporting could be considered the result of user fatigue over the duration of the protocol, engagement did not similarly decline over the study period, suggesting that participants did not lose interest in the IVDRS. This finding raises the possibility that there was a learning effect where adverse safety events truly declined as participants gained self-management abilities through the protocol. Finally, the reporting of adverse safety events relies on the participants’ judgement of what was attributable to medications and treatments, and raises the possibility of inaccurate reporting. Nevertheless, additional physician review demonstrated the vast majority of events reported could plausibly be related to medications administered at the time of the event. This review reduced the likelihood of overreporting, but did not diminish the possibility that participants may have failed to report events that could have been attributed to a medication, but they judged otherwise.

### Comparison With Prior Work

Diary methods have become increasingly used to record PROs. Use of Internet and paper-based diaries have become widely prevalent and range from monitoring of headaches, epilepsy activity, rheumatoid arthritis severity, and glucose control in diabetes [18-21]. These methods include acquisition of real-time experiences or ecological momentary assessments using end-of-day evaluations or the Day Reconstruction Method [12,13,22]. These methodologies can gauge participant sentiments, quality-of-life estimates, or symptoms as proposed here [12]. Such diaries are subject to recall bias, imprecise measurements, and potential overweighting of negative experiences [4,23].The use of newer hand-held electronic devices to record PROs needs to overcome challenges related to security, cost, and technological limitations of the target population, as are common in CKD and dialysis populations [13].

We are unaware of prior studies using diaries to record patient-related safety experiences in CKD. We elected to use a telephone-based portal, typically using a landline, to engage CKD patients in recording experiences related to symptoms or incidents they viewed as attributable to their medical treatment. This communication modality matches the technological proficiency characteristics of much of the target population [24,25]. We have previously shown that CKD patients have variable success with the use of mobile devices and Web-based applications [24,25]. We also chose a weekly scheduled call in order to balance bias anticipated from a 7-day recall period with the inconvenience of more frequent scheduled calls.

The study also demonstrates the prominence of hypoglycemia in the CKD population where diabetes is common along with the use of insulin and other diabetic treatments. However, the rate of nonhypoglycemic events with the IVDRS, as opposed to with the SKC paper diary, reveals the broader range of PROs detected with this technology. The association of several patient-reported experiences with hypoglycemia corroborates findings from a prior report linking hypoglycemia to other patient-reported safety incidents [14].

### Conclusions

We have demonstrated that an IVDRS designed to detect adverse safety events sheds light on a broader dimension of the CKD “phenotype” that is likely to be underreported in the clinical setting. The increased detection of PROs using a low-cost and simple communication technology has the potential to enhance the care of CKD patients, improve their safety, and address the high rate of poor outcomes common in this population.

## Abbreviations

CKD: chronic kidney disease
eGFR: estimated glomerular filtration rate
ER: emergency room
FDA: US Food and Drug Administration
GEE: generalized estimating equation
GFR: glomerular filtration rate
IQR: interquartile range
IVDRS: interactive voice-inquiry dial-response system
MD: medical doctor
PRO: patient-reported outcome
SF-12: 12-item Short Form Health Survey
SF-36: 36-item Short Form Health Survey
SKC: Safe Kidney Care
